# Validation of a self-administered web-based 24-hour dietary recall among pregnant women

**DOI:** 10.1186/s12884-018-1741-1

**Published:** 2018-04-23

**Authors:** Claudia Savard, Simone Lemieux, Jacynthe Lafrenière, Catherine Laramée, Julie Robitaille, Anne-Sophie Morisset

**Affiliations:** 1 École de nutrition de l’Université Laval, Pavillon Paul-Comtois, Université Laval, 2425, rue de l’Agriculture, local 2412, Québec, G1V 0A6 Canada; 2 Axe d’endocrinologie et de néphrologie du Centre de Recherche du CHU de Québec-Université Laval, Québec, QC Canada; 3 Institut sur la nutrition et les aliments fonctionnels, Université Laval, Québec, QC Canada

**Keywords:** Validation, Dietary assessment, 24-h dietary recall, Dietary intakes, Pregnancy, Pregnant women

## Abstract

**Background:**

The use of valid dietary assessment methods is crucial to analyse adherence to dietary recommendations among pregnant women. This study aims to assess the relative validity of a self-administered Web-based 24-h dietary recall, the R24W, against a pen-paper 3-day food record (FR) among pregnant women.

**Methods:**

Sixty (60) pregnant women recruited at 9.3 ± 0.7 weeks of pregnancy in Quebec City completed, at each trimester, 3 R24W and a 3-day FR. Mean energy and nutrient intakes reported by both tools were compared using paired Student T-Tests. Pearson correlations were used to analyze the association between both methods. Agreement between the two methods was evaluated using cross-classification analyses, weighted kappa coefficients and Bland-Altman analyses.

**Results:**

Pearson correlation coefficients were all significant, except for vitamin B_12_ (*r* = 0.03; *p* = 0.83) and ranged from 0.27 to 0.76 (*p* < 0.05). Differences between mean intakes assessed by the R24W and the FR did not exceed 10% in 19 variables and were not significant for 16 out of 26 variables. In cross-classification analyses, the R24W ranked, on average, 79.1% of participants in the same or adjacent quartiles as the FR.

**Conclusions:**

Compared to a 3-day FR, the R24W is a valid method to assess intakes of energy and most nutrients but may be less accurate in the evaluation of intakes of fat (as a proportion of energy intake), vitamin D, zinc and folic acid. During pregnancy, the R24W was a more accurate tool at a group-level than at an individual-level and should, therefore, be used in an epidemiological rather than a clinical setting. The R24W may be particularly valuable as a tool used in cohort studies to provide valid information on pregnant women’s dietary intakes and facilitate evaluation of associations between diet and adverse pregnancy outcomes.

**Electronic supplementary material:**

The online version of this article (10.1186/s12884-018-1741-1) contains supplementary material, which is available to authorized users.

## Background

Accurate assessment of dietary intakes is a major pillar of nutrition research, counseling and intervention [1]. The use of valid dietary assessment tools is essential to compare dietary intakes to current nutritional recommendations and to measure associations between diet and health outcomes [2, 3]. Dietary assessment is especially important during pregnancy, as inadequate or excess nutrition during this period can adversely affect both the mother and the fetus [4–6]. Considering that pregnant women’s dietary needs and intakes are influenced by numerous endo- and exogenous factors, evaluating the adequacy of diet throughout pregnancy can be complex. Physiological changes occurring in pregnancy, such as rises in blood volume, extracellular liquids, adipose tissue and placental weight, lead to an increase in dietary requirements and can heighten or suppress appetite [7, 8]. Moreover, nausea, attitudes and behaviors towards food, body image perception as well as socioeconomic status have all been known to impact pregnant women’s food intakes [9–11].

In pregnant women, as in the general population, food records (FR) are known as the gold standard for dietary assessment [12, 13]. Despite their validity, FR and other pen-paper dietary assessment methods are time consuming, both for participants and research personnel. FR are also prone to a high social desirability bias [2, 14]. For these reasons, web-based FR and web-based recall methods, such as food frequency questionnaires (FFQ) and 24-h dietary recalls, are gaining popularity. Compared to traditional/pen-paper methods, web-based methods are more cost and time effective as they can generate nutritional data automatically and are less prone to data entry errors [15–17]. It has also been reported that web-based tools tend to enhance completion rates by reducing the burden associated with pen-paper dietary assessment methods [18]. For example, contrary to FR, FFQ and dietary recalls are less time consuming because they do not require the participant to weigh or measure every food item consumed. [19]. On the other hand, both FFQ and dietary recalls are subject to memory bias, as participants are asked to report their dietary intakes retrospectively [20]. Moreover, FFQ are more often used to evaluate diets over longer periods of time (e.g. 1 to 12 months), as opposed to dietary recalls, which generally assess short-term dietary intakes [2, 19]. Therefore, in a context like pregnancy, where various factors can impact appetite on a daily basis, using multiple 24-h dietary recalls might provide the most accurate estimate of pregnant women’s dietary intakes [4, 21].

Although web-based 24-h dietary recalls are now more frequent in epidemiological studies, very few have been validated in a pregnant population [22]. Since the validation of a dietary assessment tool in the target population is a necessary first step prior to interpreting and generalizing data [23, 24], this study aims to assess the relative validity of a self-administered web-based 24-h dietary recall (R24W) against a 3-day FR among a population of French Canadian pregnant women. We hypothesize that the R24W is a valid tool to assess dietary intakes in pregnant women.

## Methods

### Participants

Eighty-six (86) pregnant women recruited from April 2016 to May 2017 at the CHU de Québec – Université Laval (Québec, Canada) were included in the ANGE project (*Apports Nutritionnels Durant la Grossesse*). The general aim of the ANGE study was to evaluate dietary intakes throughout pregnancy in association with nutritional recommendations, gestational weight gain, glucose tolerance and metabolic profile. Women younger than 18 years old and with a gestational age greater than 12 weeks of pregnancy at the time of recruitment were excluded. Women with a previously diagnosed disease affecting their metabolic profile and/or their dietary intakes (i.e. Type 1 and type 2 diabetes, renal disease, inflammatory and immunity disorders) were also excluded. For the validation analyses, our final sample was restricted to women who completed both the 3FR and the R24W on three occasions within each trimester (*n* = 60). The ANGE project was approved by the CHU de Québec – Université Laval Research Center’s Ethical Committee and participants gave their informed written consent at their first visit to the research center.

### The automated web-based 24-h recall (R24W)

In the 1^st^ (range: 7-14 weeks), 2^nd^ (range: 20-27 weeks) and 3^rd^ (range: 31-38 weeks) trimesters of pregnancy, each participant was asked to complete a total of 3 R24W on 2-week days and 1-weekend day (total of 9 R24W throughout pregnancy). The development of the R24W has been previously described [25]. Briefly, the R24W uses a sequence of questions inspired by the USDA’s Automated Multiple Pass Method (AMPM) [26]. In contrast to the AMPM, the R24W uses a meal-based approach in its 1st step. The application also sends automatic e-mails on randomly chosen dates to remind the participants to complete the R24W. Participants are required to watch a mandatory tutorial video prior to their first R24W. The database includes 2865 food items that are linked to the Canadian Nutrient File [27] to enable automatic extraction of nutrient values. Participants can report an unlimited number of meals and snacks for a 24-h period. The database also includes a total of 687 recipes created to ease the entry of mixed dishes (e.g. spaghetti with meat sauce, salads, etc.). Pictures depicting multiple portion sizes with corresponding units and/or volume are available for more than 80% of all food items. After selecting a food item, participants must choose the picture that best represents the amount of food eaten. In addition, systematic questions are asked about frequently forgotten food items including toppings, condiments, fats, snacks and drinks. The R24W performed well in a validation study where the real dietary intakes of 62 non pregnant adults were known [28] and a pre-test revealed that 29 adults found the R24W easy to understand and complete [25].

### 3-day food record

At baseline (range: 7-14 weeks), in the 2^nd^ trimester (range: 20-27 weeks) and in the 3^rd^ trimester (range: 31-38 weeks), participants were given a three 3-day FR to complete on 2-week days and 1-weekend day (total of 3 FR throughout pregnancy). A trained dietician gave, at baseline, detailed explanations to each participant on how to complete their FR. Written instructions, a list of frequently forgotten food items and an example of a correctly completed FR were also provided to the participants. Participants were not required to weigh the foods consumed but were asked to measure their food with household measurements (cups, teaspoons) and indicate a commercial product’s brand when applicable. Participants were also asked to provide detailed information about mixed dishes consumed, either by writing, printing, or scanning a recipe from a website or a cookbook. Women were highly encouraged to preserve their normal eating habits when completing the FR. When participants gave back their FR, completeness and portion sizes were reviewed by the same or another trained dietician.

### Nutritional analyses

In each trimester, the R24W and FR were completed on different days. This was established because the R24W requires a participant to recall the foods consumed on the previous day and thus, having completed a FR for the same day would help a participant remember each food eaten. All food items were coded using the 2015 version of the Canadian Nutrient File [27], either automatically, for the R24W, or with Nutrific software (Laval University, Quebec) for the FR. Data from FR was analyzed and initially coded by a research assistant. A trained dietician reviewed data entry and coding for mistakes or omissions. Data for energy and 22 nutrients were analyzed for both the FR and the R24W. Dietary supplement intakes were not considered in the analyses.

### Other variables

Pre-pregnancy body weight was self-reported and height was measured at baseline to calculate pre-pregnancy BMI. Web questionnaires regarding pregnancy related variables (i.e. presence of nausea, vomiting, food cravings, etc.) were completed at each trimester and a socio-economic Web questionnaire was completed once during pregnancy.

### Statistical analyses

At each trimester, means and standard deviations for energy, macro- and micronutrient intakes as well as percentage of energy from carbohydrates (% carbohydrates), fat (% fat) and proteins (% proteins) were calculated from the 3-day FR and the 3 R24W. Paired Student t-tests were performed to assess the difference between mean intakes reported by the R24W and mean intakes reported by the FR. Percent differences between the 3 R24W and the 3-day FR [(Mean R24W – Mean FR) / Mean FR)] were also calculated. Pearson correlations were used to evaluate the associations between nutrient intakes from the R24W and the FR. Non-normally distributed variables were log or box-cox transformed. Cross-classification analyses were performed to examine the ability of the R24W to classify participants into similar or adjacent quartiles of dietary intakes classified by the FR. Weighted kappa scores were calculated to assess whether or not the results of the cross-classification analyses were attributable to chance only. The Bland-Altman analysis, described as the Spearman correlation between the mean of both tools [(Mean R24W + Mean FR) / 2] and the difference between both tools (Mean FR – Mean R24W) for each participant, was used. Differences and correlations were considered statistically significant at *p* <  0.05. Finally, all tests (Paired student t-test, percent difference, Pearson correlation, cross-classification and misclassification, weighted kappa score and Bland-Altman analysis) were pooled to obtain a seven-criteria validity analysis, according to the classification suggested by Lombard et al. [29]. A good (G), acceptable (A) or poor (P) score was attributed to each variable for all 7 criteria. Statistical analyses were performed in JMP version 12 (SAS Institute Inc., NC, USA), with the exception of the weighted kappa score, which was calculated with SAS version 9.4 (SAS Institute Inc., NC, USA).

## Results

Characteristics of the participants are presented in Table 1. Our sample included 60 pregnant women with a mean age of 32.5 ± 3.5 years old and an average pre-pregnancy BMI of 25.3 ± 5.8 kg/m^2^. Ninety-eight percent of our participants were Caucasian and 81.7% had completed a university degree. In the 1st trimester of pregnancy, almost all participants (86.7%) reported nauseous symptoms, as opposed to the 2nd and 3rd trimesters, where much smaller proportions of women reported such symptoms.

**Table 1:** Participants’ characteristics (*N* = 60)

Variables	Mean ± SD or N (%)
Age (years)	32.5 ± 3.5
BMI (kg/m^2^)	25.3 ± 5.8
Ethnicity – Caucasian	59 (98.3)
Education	
High school	2 (3.3)
College	9 (15.0)
University	49 (81.7)
Household income^a^	
< 40,000 $	3 (5.0)
40,000 – 59,999 $	8 (13.3)
60,000 – 79,999 $	10 (16.7)
80,000 – 99,999 $	13 (21.7)
> 100,000 $	25 (41.7)
Gestational age at baseline (weeks)	9.3 ± 0.7
Number of children	
None^b^	22 (36.7)
1	33 (55.0)
2	4 (6.7)
3	1 (1.7)
Experienced nausea	
1^st^ trimester	52 (86.7)
2^nd^ trimester	20 (33.3)
3^rd^ trimester	14 (23.3)

^a^For *N* = 59; ^b^Pregnant with their first child

Five women completed 8 of the 9 required R24W and 2 additional women partially completed one of their 3 FR (2 out of 3 days). We did not exclude these women from our statistical analyses because including them did not affect our results. Completion statistics for the R24W are presented in Table 2. Average R24W completion time significantly decreased at each trimester (*p* < 0.001) and the number of items reported was significantly lower in the 3rd compared to the 2nd trimester (*p* = 0.02).

**Table 2:** Completion statistics of the R24W

	Mean (SD)			*P* value
	1st trimester	2nd trimester	3rd trimester	
Completion time (minutes)	21.8 (7.5)	19.2 (6.8)	16.3 (5.1)	< 0.0001
Reported meals (N)	5.1 (0.9)	4.8 (1.1)	5.0 (1.2)	0.13
Reported items (N)	20.5 (4.0)	20.3 (4.1)	19.4 (4.0)	0.025

In order to lighten the figures and tables shown in the results section of this article, only 2nd trimester validity analyses are presented. Results from the 1st and 3rd trimesters are available in the supplementary material, and some of these results are briefly presented throughout the section below. Overall, 2nd trimester results were representative of the association and agreement between the R24W and the FR throughout pregnancy, as they were statistically stronger than the 1st but not the 3rd trimester results.

Table 3 presents differences between the data from R24W and the FR for the 2nd trimester, as well as Pearson correlation coefficients. Mean intakes of energy, fat, % fat, saturated fatty acids (SFA), cholesterol, vitamin C, vitamin D, magnesium, phosphorus, zinc and calcium were significantly different between both tools (average difference: 12.2%; *p* < 0.05). Results were similar in other trimesters (Additional file 1: Table S1), as differences between mean R24W and FR intakes were significant for 8 variables in both the 1st (energy, fat, SFA, riboflavin, vitamin D, phosphorus, calcium and sodium; average difference: 13.0%) and 3rd trimesters (% fat, SFA, cholesterol, vitamin B_6_, vitamin D, magnesium, phosphorus and calcium; average difference: 11.4%). In the 2nd trimester, the highest percent differences were observed for vitamin D (20.6%; *p* = 0.02) and calcium (21.6%; *p* = 0.0003). Pearson correlation coefficients in the 2nd trimester ranged from 0.27 to 0.76 and were all significant with the exception of vitamin B_12_ (*r* = 0.03; *p* = 0.83). Similarly, all 3rd trimester correlations were significant, but non-significant correlations were observed for % fat (*r* = 0.09; *p* = 0.50), folic acid (*r* = 0.20; *p* = 0.13) and vitamin B_12_ (*r* = 0.19; *p* = 0.14) in the 1st trimester.

**Table 3:** Differences between mean dietary intakes reported by the R24W and the FR in the 2nd trimester

	R24W (SD)	FR (SD)	% difference	Pearson correlation
Energy (kcal)	2357 (489)	2239 (506)	5.3^*^	0.68^*^
Carbohydrates (g)	286.7 (67.7)	279.1 (76.2)	2.7	0.76^*^
Fat (g)	94.3 (26.1)	84.9 (22.6)	11.1^*^	0.60^*^
Proteins (g)	99.6 (20.1)	98.5 (22.6)	1.1	0.37^*^
% Carbohydrates	48.7 (5.4)	49.7 (6.0)	−2.0	0.53^*^
% Fat	35.8 (4.9)	34.1 (4.8)	5.0^*^	0.48^*^
% Proteins	17.2 (3.0)	17.8 (3.1)	−3.4	0.58^*^
SFA (g)	34.7 (11.7)	30.3 (10.4)	14.5^*^	0.48^*^
Cholesterol (mg)	289.4 (95.2)	322.6 (134.4)	−10.3	0.29^*^
Vitamin A (μg)	943.6 (412.4)	932.0 (630.3)	1.2	0.31^*^
Thiamin (mg)	2.0 (0.6)	1.9 (0.6)	5.3	0.68^*^
Riboflavin (mg)	2.4 (0.6)	2.3 (0.7)	4.3	0.46^*^
Niacin (mg)	26.7 (5.7)	27.2 (9.3)	−1.8	0.48^*^
Vitamin B6 (mg)	1.9 (0.5)	1.9 (0.6)	0	0.44^*^
Folic Acid (μg)	400.5 (100.8)	405.9 (144.1)	−1.3	0.54^*^
Vitamin B12 (μg)	5.5 (2.6)	5.1 (2.6)	7.8	0.03
Vitamin C (mg)	139.9 (85.6)	162.0 (83.9)	−13.6^*^	0.69^*^
Vitamin D (IU)	272.0 (132.6)	225.6 (131.4)	20.6^*^	0.27^*^
Magnesium (mg)	398.9 (100.4)	359.3 (113.9)	11.0^*^	0.57^*^
Phosphorus (mg)	1678.1 (370.8)	1557.1 (384.4)	7.8^*^	0.43^*^
Zinc (mg)	13.3 (3.1)	11.9 (3.0)	11.8^*^	0.28^*^
Iron (mg)	15.9 (4.7)	15.9 (4.9)	0	0.58^*^
Calcium (mg)	1372.3 (500.0)	1128.3 (447.0)	21.6^*^	0.51^*^
Potassium (mg)	3302.9 (826.4)	3326.1 (848.5)	−0.7	0.62^*^
Sodium (mg)	3322.0 (891.3)	3088.9 (1038.3)	7.5	0.51^*^
Dietary fibers (g)	24.8 (8.2)	24.2 (6.8)	2.5	0.71^*^
Average			6.7	0.50

^*^T-test and Pearson correlation coefficients with a *P* value < 0.05

The R24W classified 79.1% (range 68.3-90.0%) of the participants in the same or adjacent quartile compared with the FR in the 2^nd^ trimester (Table 4). Furthermore, misclassification, i.e. when a participant is classified in the 1^st^ quartile by one tool and in the 4^th^ by the other, occurred in 3.9% (range 0-10.0%) of all cases in the 2^nd^ trimester. Cross-classification analyses revealed similar results in the 1^st^ and 3^rd^ trimesters (Additional file 2: Table S2). Weighted kappa scores ranged from 0.09 to 0.49 (average 0.32) in the 2^nd^ trimester, with the lowest value being for cholesterol. Significant Spearman correlations between the mean of both tools and the difference between both tools (proportional bias) were observed for cholesterol and niacin in the 2nd trimester (Table 5). A proportional bias was also observed for thiamin and vitamin C as well as for carbohydrates, % carbohydrates, % fat, thiamin, niacin, vitamin B6, folic acid and fibers in the 1st and 3rd trimesters, respectively (Additional file 3: Table S3).

**Table 4:** Cross-classification of intakes by quartiles and weighted kappa coefficient in the 2nd trimester

	%				Weighed Kappa
	Same quartile	Adjacent quartiles	± 1 Quartile apart	Misclassification (quartile 1 vs 4)	
Energy	43.3	38.3	81.6	1.7	0.39
Carbohydrates	53.3	33.3	86.6	3.3	0.49
Fat	40.0	41.7	81.7	1.7	0.36
Proteins	40.0	38.3	78.3	5.0	0.31
% Carbohydrates	41.7	38.3	80.0	5.0	0.33
% Fat	33.3	45.0	78.3	1.7	0.28
% Proteins	51.7	35.0	86.7	1.7	0.49
Saturated fatty acids	38.3	43.3	81.6	6.7	0.31
Cholesterol	21.7	46.7	68.4	3.3	0.09
Vitamin A	30.0	41.7	71.7	1.7	0.20
Thiamin	48.3	38.3	86.7	1.7	0.47
Riboflavin	33.3	51.7	85.0	1.7	0.33
Niacin	26.7	41.7	68.4	1.7	0.15
Vitamin B6	38.3	35.0	73.3	8.3	0.23
Folic Acid	40.0	33.3	73.3	3.3	0.28
Vitamin B12	30.0	38.3	68.3	8.3	0.12
Vitamin C	40.0	50.0	90.0	0	0.44
Vitamin D	33.3	38.3	71.6	8.3	0.17
Magnesium	50.0	38.3	88.3	5.0	0.47
Phosphorus	40.0	38.3	78.3	1.7	0.33
Zinc	31.7	40.0	71.7	10.0	0.15
Iron	43.3	36.7	80.0	3.3	0.36
Calcium	40.0	48.3	88.3	1.7	0.41
Potassium	41.7	33.3	75.0	3.3	0.31
Sodium	48.3	31.7	80.0	8.3	0.36
Dietary fibres	46.7	36.7	83.4	3.3	0.41
Average	39.4	39.7	79.1	3.9	0.32

**Table 5:** Seven criteria validity analysis of the R24W in the 2^nd^ trimester

	Individual level			Group level			Total of poor outcomes
	Association	Agreement		Agreement		Presence of bias	
	Pearson coefficient	Cross-classification	Kappa score	% difference	T-test	Bland- Altman	
Criteria for good (G) outcome	≥0.50	≥ 50% in same quartile; < 10% in opposite quartile	≥0.61	0-10.9%	*P* > 0.05	P > 0.05	
Criteria for acceptable (A) outcome	0.20-0.49		0.20-0.60	11.0-20%			
Criteria for poor (P) outcome	< 0.20	< 50% in same quartile; ≥10% in opposite quartile	< 0.20	> 20%	*P* ≤ 0.05	*P* ≤ 0.05	
Energy	G	P-G	A	G	P	G	2
Carbohydrates	G	G-G	A	G	G	G	0
Fat	G	P-G	A	A	P	G	2
Proteins	A	P-G	A	G	G	G	1
% Carbohydrates	G	P-G	A	G	G	G	1
% Fat	A	P-G	A	G	P	G	2
% Proteins	G	G-G	A	G	G	G	0
SFA	A	P-G	A	A	P	G	2
Cholesterol	A	P-G	P	G	G	P	3
Vitamin A	A	P-G	A	G	G	G	1
Thiamin	G	P-G	A	G	G	G	1
Riboflavin	A	P-G	A	G	G	G	1
Niacin	A	P-G	P	G	G	P	3
Vitamin B6	A	P-G	A	G	G	G	1
Folic Acid	G	P-G	A	G	G	G	1
Vitamin B12	P	P-G	P	G	G	G	3
Vitamin C	G	P-G	A	A	P	G	2
Vitamin D	A	P-G	P	P	P	G	4
Magnesium	G	G-G	A	A	P	G	1
Phosphorus	A	P-G	A	G	P	G	2
Zinc	A	P-P	P	A	P	G	4
Iron	G	P-G	A	G	G	G	1
Calcium	G	P-G	A	P	P	G	3
Potassium	G	P-G	A	G	G	G	1
Sodium	G	P-G	A	G	G	G	1
Dietary fibres	G	P-G	A	G	G	G	1
Total of poor outcomes	1	23-1	5	2	10	2	44
Average							1.7

A summary of all agreement and association analyses conducted in the 2nd trimester is presented in Table 5, based on the classification suggested by Lombard et al. [29]. In the 2nd trimester, the number of poor outcomes by variable ranged from 0 (carbohydrates and % protein) to 4 (vitamin D and zinc). In the 1st trimester, % fat, vitamin D and folic acid were the variables with the highest number of poor outcomes (*n* = 4), and all 3rd trimester variables accumulated less than 4 poor outcomes each (Additional file 3: Table S3).

## Discussion

This is the first study to assess the relative validity of a web-based 24-h dietary recall in comparison with a 3-day FR among a population of pregnant women in all trimesters. All agreement and association analyses showed that, for most nutrients, the R24W can provide an estimation of pregnant women’s dietary intakes that is similar to the one obtained with a 3-day FR. Our results demonstrate that the R24W can be used to evaluate dietary intakes throughout pregnancy. To our knowledge and as mentioned by Vézina-Im et al. (2014) [30], there is a lack of current literature relevant to the use of this type of tool. Therefore, our analyses must be compared with those of studies that validated web-based 24-h dietary recalls against a FR in non-pregnant adult populations.

Overall, Pearson correlations between both tools assessed in the 2^nd^ trimester of pregnancy have shown good associations. Indeed, our results revealed that 14 nutrients had a correlation coefficient above or equal to 0.50 which Masson et al. (2003) [31] identified as the minimal recommended value to qualify a correlation as good. Correlations of this magnitude are, however, expected when two distinct measurement methods are used to assess the same variables (e.g. vitamin D measured by the R24W vs measured by a 3-day FR) [23, 32, 33]. Our results are similar to those of Frankenfeld et al. (2012) [34], who compared 2 web-based 24-h dietary recalls (ASA24) with a 4-day dietary record in 93 non pregnant Americans and found correlation coefficients that ranged from 0.06 to 0.76. Similarly, Timon et al. (2017) [35] compared 2 web-based 24-h dietary recalls (Foodbook24) with a 4-day FR among 40 non pregnant adults and observed correlation coefficients ranging from 0.32 to 0.75, which is also comparable to our results.

In our study, we observed a correlation coefficient of 0.03 (*p* = 0.83) for vitamin B_12_ intakes in the 2^nd^ trimester, indicative of a weak association between the FR and the R24W for this nutrient. Nevertheless, this lack of association does not necessarily mean that the R24W cannot assess vitamin B_12_ intakes accurately. In fact, mean intakes of vitamin B_12_ reported by the R24W and the FR did not differ significantly in the 2^nd^ trimester (5.5 ± 2.6μg with the R24W vs 5.1 ± 2.6μg with the FR; *p* = 0.46), as well as in the 1^st^ and 3^rd^ trimesters. Moreover, in all trimesters, no proportional bias was observed for vitamin B_12_ intakes. Similarly, even though Frankenfeld et al. (2012) [34] observed a lower correlation coefficient (*r* = 0.06) for vitamin E intakes reported by the ASA24 and the FR, they found no significant difference between vitamin E intakes reported by both tools. Likewise, Comrie et al. (2009) [36], who compared a web-based food recall checklist (FoRC) with a 4-day FR among 53 University students, observed a weaker correlation (*r* = 0.30) between intakes of % fat reported by both tools, but the mean difference between both tools was not significant. Thus, in the present study, we interpret the weak association between both tools for one particular nutrient as a justification to conduct additional tests, such as paired student-t test, cross-classification and Bland-Altman analysis, but not as a justification to invalidate the data [29].

For the majority of nutrients, no significant differences were detected between mean intakes reported by the R24W and the FR, in the 2nd trimester. However, some significant differences were observed for total energy, fat, % fat, SFA, vitamin C, vitamin D, magnesium, phosphorus, zinc and calcium. For these nutrients, average difference between intakes reported by the FR and the R24W was 12.2%, which is considered as an acceptable gap according to Lombard et al. (2015) [29]. Overall, intakes reported by the R24W were higher than those reported by the FR for 17 out of 26 variables. Similar results were obtained by Timon et al. (2017) [35], where significant differences between the Web-based 24HR and the FR were observed in intakes of % fat, protein, dietary fibers, riboflavin, iron, potassium and sodium. In comparison, De Keyzer et al. (2011) [37] compared two computer assisted 24-h recalls (24HR) with a 5-day FR and found significant differences between intakes of energy and 8 nutrients (fat, fatty acids, cholesterol, alcohol, vitamin C, thiamine, riboflavin and iron) reported by both tools. Furthermore, De Keyzer et al. (2011) [37] also observed higher intakes with the 24HR than with the FR and suggested it might be due to portion size estimation by food photographs. In fact, although photographs are generally useful to accurately recall portion size, some studies have mentioned considerable over and/or underestimations of the real amount of food eaten when participants were asked to use photographs to recall their food intakes [38–40]. On the other hand, a previous validation study comparing the R24W with known dietary intakes found that the self-reported portion sizes were, on average, only 3.2 g higher than the real portion sizes offered to participants [28]. However, the same study observed that portion sizes smaller than 100 g were significantly overestimated by 17.1%. This could partially explain why, in our study, energy, fat, % fat and SFA intakes were higher when reported by the R24W, as smaller portioned food items include fats, sauces, toppings and cheese [28]. Significantly higher intakes observed with the R24W could also be explained by the presence of social desirability and reactivity bias, both frequently observed with the FR [2, 14]. Therefore, it is possible that our participants had an increased tendency to underestimate and/or underreport portion sizes when completing the FR in comparison to when they completed the R24W.

Cross-classification analyses in the 2nd trimester revealed an acceptable agreement on an individual-level between the R24W and the FR. Classification of participants in the same and in the same or adjacent quartiles averaged 39.4% (range: 21.7-53.3%) and 79.1% (range: 68.3-90.0%), respectively. These results are similar to those observed by Frankelfeld et al. (2012) [34] and Timon et al. (2017) [35] in which ranking of participants in the same or adjacent quartiles ranged from 62.6% to 79.8% and from 69.2% to 92.3%, respectively. Moreover, in our study, only zinc was characterized by a gross misclassification (opposing quartiles) of more than 10% of participants. This is especially important since the ability of a method to accurately rank participants according to their dietary intakes is essential in the evaluation of diet-disease associations [23]. Furthermore, weighted kappa scores averaged 0.32, thus representing an acceptable agreement according to the terminology of Lombard et al. [29] and indicating that the ranking of participants in the same or adjacent quartiles was not attributable to chance [29]. It is important to mention that a perfect agreement between the R24W and the FR was very unlikely to be observed, considering that both tools did not evaluate dietary intakes on the same 3 days. Moreover, since within-person day-to-day variability is high for both 24-h dietary recalls and FR and since both tools were completed on 3 different days, the ranking of participants is complex [41]. Day-to-day variability may be further enhanced during pregnancy, as pregnant women’s dietary intakes may be influenced by nausea and vomiting. [42]. It is possible that some women experienced more nausea and vomiting during the 3 days they completed the FR compared to the 3 R24W days, or vice-versa. Thus, a woman could have been ranked in the 1st quartile according to her intakes reported by one tool and in the 3rd or 4th quartile according to her intakes reported by the other tool. For these reasons, weighted kappa coefficients and the results of cross-classification analyses should be interpreted with caution and in combination with the other validity analyses conducted in this study.

As previously mentioned, analyses conducted in the 3rd trimester were statistically stronger when compared to those of the 1st and 2nd trimesters, thus suggesting a greater relative validity in the 3rd trimester. This might be explained, in part, by participant’s increased experience and comfort with completing both tools towards the end of their pregnancy. By the third trimester, participants had completed six R24W and two 3-day FR. This could also partially explain the significant decrease in completion time across trimesters of pregnancy. Moreover, fewer participants reported experiencing nausea in the 3rd trimester, which may also explain why there were less variations between dietary intakes recorded by the last R24W and FR, compared with the 1st and 2nd trimesters. Since our study is the first to assess the validity of a web-based 24-h recall in all trimesters of pregnancy, we were not able to compare our results to previous literature and these suppositions should be interpreted with caution.

In the 2nd trimester, only 2 (vitamin D and zinc) out of 26 variables accumulated a total of 4 poor association and agreement outcomes. Similar results were observed in the 1st (% fat, folic acid and vitamin D) and 3rd trimesters. Lombard et al. (2015) [29] suggested that nutrients with the highest number of poor outcomes, %fat, folic acid, vitamin D and zinc in our results, might not be consumed on a daily basis by the studied population and would be better assessed by a FFQ rather than a R24W or a FR. This is of particular interest considering that folic acid is essential during pregnancy and plays an important role in fetal growth and development [43, 44]. Moreover, deficiencies in folic acid are associated with higher risk of birth defects, particularly neural tube defects [44]. Thus, an inaccurate intake estimation of this nutrient could be harmful, especially among pregnant women that are less compliant with their prenatal dietary supplements [45]. The combined use of a FFQ with the R24W and even biomarker analyses should, therefore, be considered to accurately assess dietary intakes of folic acid, vitamin D and zinc [29]. Globally, better results were observed with the group-level analyses (paired Student t-test and Bland-Altman analysis) in comparison with individual validity outcomes (Pearson correlation, cross-classification analysis and weighted kappa coefficient). This greater validity on a group-level in comparison to individual-level was also observed by Comrie et al. (2009) [36], although this study did not use all 7 statistical analyses conducted in the present study. However, the accuracy of dietary assessment at an individual level (i.e. when the assessed intakes of an individual accurately reflects real intakes) is not essential to provide valid and useful data on nutrition and health outcomes [41]. Therefore, the results of our group-level analyses suggest that the R24W is a valid tool to assess average dietary intakes of pregnant women but should be used with caution when counselling dietary changes to pregnant women in a clinical setting.

To our knowledge, this is the first dietary assessment validation study among pregnant women to compare a web-based 24-h recall with a FR by using seven association and agreement analyses for each pregnancy trimester. This summary analysis allowed a more in depth understanding of the accuracy and precision of the R24W, as well as an identification of the R24W’s strengths and limitations. It can be argued that, for a better estimate of usual dietary intakes, additional R24W and FR days would have been needed. Yet, asking our participants to recall and report their dietary intakes for more than 6 days per trimester could have worsened compliance, participation rate, and potentially altered our results. Our validation study also has limited generalizability because our study population was highly educated and almost 100% Caucasian. In addition, our small study sample (*n* = 60) might have attenuated the strength of some of our statistical analyses, e.g. correlation coefficients and cross-classification. Despite our small study sample, our results showed that the R24W was a relatively valid tool.

## Conclusions

In summary, we observed that compared to the FR, the R24W has good relative validity to assess energy and most nutrients in all trimesters. The R24W did not, however, perform as well for the assessment of intakes of % fat, vitamin D, folic acid and zinc. The use of combined dietary assessment methods, including FFQs and biomarker analyses, is, therefore, recommended to ensure an accurate estimation of dietary intakes for these nutrients. The R24W demonstrated a greater group-level validity compared to an individual-level validity and should, therefore, be used in an epidemiological rather than a clinical setting. Nevertheless, in addition to being less expensive and more effective than pen-paper methods, the R24W has the potential to provide accurate and precise information on pregnant women’s dietary intakes, and, therefore allows the evaluation of potential associations between dietary intakes and adverse pregnancy outcomes.

## Additional files

Additional file 1: **Table S1.** Differences between mean dietary intakes reported by the R24W and the FR in the 1^st^ and 3^rd^ trimesters. This additional file presents differences (Percentage differences and results of Student T-test) between mean dietary intakes reported with the R24W and the FR in the 1st and 3rd trimesters as well as Pearson correlation coefficients. (DOCX 22 kb)

Additional file 2: **Table S2.** Cross-classification of intakes by quartiles and weighted kappa coefficient in the 1^st^ and 3^rd^ trimesters. This additional file presents the proportion of participants whose dietary intakes reported by both tools in the 1st and 3rd trimesters were ranked in the same, adjacent and opposite quartiles. (DOCX 20 kb)

Additional file 3: **Table S3.** Seven criteria validity analysis of the R24W in the 1^st^ and 3^rd^ trimesters. This additional file presents a summary of all agreement and association analyses conducted in the 1st and 3rd trimesters. (DOCX 21 kb)

## Abbreviations

% carbohydrates: Percentage of total energy from carbohydrates
% fat: Percentage of total energy from fat
% proteins: Percentage of total energy from proteins
24HR: 24-h recall
AMPM: Automated Multiple-Pass Method
ANGE: Apports Nutritionnels durant la GrossessE (Nutritional intakes during pregnancy)
ASA24: Automated Self-Administered Recall System
BMI: Body mass index
CHU: University-affiliated Hospital Center
FFQ: Food frequency questionnaire
FR: Food record
R24W: The automated self-administered 24-h dietary recall developed by Laval University
SFA: Saturated fatty acids
USDA: United States Department of Agriculture
