# Supplementary material for: Validation of a self-administered web-based 24-hour dietary recall among pregnant women
Source: BMC Pregnancy Childbirth. 2018 Apr 23;18:112. doi: 10.1186/s12884-018-1741-1 (PMC5913813; doi:10.1186/s12884-018-1741-1)
Supplement: Supplementary file 1 — Table S1. Differences between mean dietary intakes reported by the R24W and the FR in the 1st and 3rd trimesters. This additional file presents differences (Percentage differences and results of Student T-test) between mean dietary intakes reported with the R24W and the FR in the 1st and 3rd trimesters as well as Pearson correlation coefficients. (DOCX 22 kb) [file 12884_2018_1741_MOESM1_ESM.docx]

**Additional file 1: Table S1: Differences between mean dietary intakes reported by the R24W and the FR in the 1^st^ and 3^rd^ trimesters.**

|  | **R24W (SD)** | **FR (SD)** | **%**  **difference** | **Pearson correlation** |
| --- | --- | --- | --- | --- |
| ***1^st^ trimester*** |  |  |  |  |
| Energy (kcal) | 2313 (472) | 2180 (398) | 6.1* | 0.48* |
| Carbohydrates (g) | 284.3 (69.4) | 273.2 (55.1) | 4.1 | 0.45* |
| Fat (g) | 90.5 (20.5) | 82.5 (23.0) | 9.7* | 0.35* |
| Proteins (g) | 98.8 (20.2) | 95.3 (19.3) | 3.7 | 0.60* |
| % Carbohydrates | 49.0 (5.0) | 50.3 (6.1) | -2.6 | 0.30* |
| % Fat | 35.3 (4.1) | 33.7 (5.0) | 4.7 | 0.09 |
| % Proteins | 17.2 (2.5) | 17.6 (3.0) | -2.3 | 0.48* |
| Saturated fatty acids (g) | 33.0 (8.0) | 29.3 (8.45) | 12.6* | 0.44* |
| Cholesterol (mg) | 301.7 (96.7) | 286.7 (107.4) | 5.2 | 0.40* |
| Vitamin A (μg) | 897.2 (302.3) | 893.4 (396.8) | 0.4 | 0.33* |
| Thiamin (mg) | 1.8 (0.6) | 1.9 (0.7) | -5.3 | 0.42* |
| Riboflavin (mg) | 2.4 (0.6) | 2.2 (0.6) | 9.1* | 0.51* |
| Niacin (mg) | 27.3 (7.3) | 26.3 (7.4) | 3.8 | 0.40* |
| VitB6 (mg) | 1.9 (0.5) | 1.9 (0.6) | 0 | 0.47* |
| Folic Acid (μg) | 408.0 (97.0) | 396.1 (106.1) | 3.0 | 0.20 |
| Vitamin B12 (μg) | 5.0 (1.6) | 4.8 (2.7) | 4.2 | 0.19 |
| Vitamin C (mg) | 161.8 (64.4) | 168.7 (83.9) | -4.1 | 0.46* |
| Vitamin D (IU) | 233.2 (103.7) | 193.9 (109.0) | 20.3* | 0.51* |
| Magnesium (mg) | 392.3 (103.0) | 365.7 (96.5) | 7.3 | 0.36* |
| Phosphorus (mg) | 1661.2 (376.5) | 1535.3 (331.1) | 8.2* | 0.64* |
| Zinc (mg) | 12.9 (3.3) | 12.0 (3.4) | 7.5 | 0.42* |
| Iron (mg) | 15.4 (4.7) | 15.0 (3.6) | 2.7 | 0.37* |
| Calcium (mg) | 1325.6 (367.6) | 1101.7 (329.1) | 20.3* | 0.50* |
| Potassium (mg) | 3292.7 (695.9) | 3373.0 (839.7) | -2.4 | 0.43* |
| Sodium (mg) | 3457.4 (911.9) | 2942.8 (834.3) | 17.5* | 0.45* |
| Fibres (g) | 24.0 (7.0) | 24.2 (7.5) | -0.8 | 0.50* |
| Average |  |  | 6.5 | 0.41 |
| ***3^rd^ trimester*** |  |  |  |  |
| Energy (kcal) | 2280 (518) | 2262 (428) | 0.8 | 0.61* |
| Carbohydrates (g) | 274.9 (60.9) | 279.2 (87.3) | -1.5 | 0.70* |
| Fat (g) | 90.7 (22.6) | 85.7 (24.0) | 5.8 | 0.47* |
| Proteins (g) | 100.8 (22.2) | 102.5 (24.5) | -1.7 | 0.62* |
| % Carbohydrates | 48.2 (5.4) | 49.1 (7.5) | -1.8 | 0.60* |
| % Fat | 35.6 (4.3) | 34.0 (5.8) | 4.7* | 0.42* |
| % Proteins | 17.9 (3.4) | 18.5 (4.2) | -3.2 | 0.63* |
| Saturated fatty acids (g) | 34.3 (9.1) | 29.6 (10.3) | 15.9* | 0.52* |
| Cholesterol (mg) | 292.4 (116.0) | 322.6 (133.0) | -9.4* | 0.57* |
| Vitamin A (μg) | 936.5 (419.1) | 972.7 (759.7) | -3.7 | 0.43* |
| Thiamin (mg) | 1.8 (0.7) | 1.9 (0.8) | -5.3 | 0.61* |
| Riboflavin (mg) | 2.6 (0.7) | 2.5 (0.8) | 4.0 | 0.64* |
| Niacin (mg) | 26.7 (6.6) | 28.3 (9.2) | -5.7 | 0.53* |
| VitB6 (mg) | 1.8 (0.4) | 2.0 (0.7) | -10.0* | 0.49* |
| Folic Acid (μg) | 400.9 (99.3) | 414.0 (151.3) | -3.2 | 0.56* |
| Vitamin B12 (μg) | 5.8 (2.7) | 5.9 (4.0) | -1.7 | 0.42* |
| Vitamin C (mg) | 137.2 (70.3) | 142.9 (79.2) | -4.0 | 0.60* |
| Vitamin D (IU) | 285.0 (159.9) | 246.7 (135.8) | 15.5* | 0.40* |
| Magnesium (mg) | 405.8 (104.1) | 380.8 (124.9) | 6.6* | 0.71* |
| Phosphorus (mg) | 1729.4 (444.4) | 1630.8 (446.9) | 6.1* | 0.67* |
| Zinc (mg) | 13.4 (3.7) | 12.4 (3.2) | 8.1 | 0.41* |
| Iron (mg) | 15.0 (3.8) | 15.9 (6.2) | 5.7 | 0.56* |
| Calcium (mg) | 1479.5 (512.2) | 1201.8 (499.0) | 23.1* | 0.68* |
| Potassium (mg) | 3278.9 (799.6) | 3413.2 (924.5) | -3.9 | 0.54* |
| Sodium (mg) | 3173.6 (898.2) | 3095.3 (1013.0) | 2.5 | 0.61* |
| Fibres (g) | 24.1 (6.5) | 24.7 (8.5) | -2.4 | 0.68* |
| Average |  |  | 6.0 | 0.56 |
