# Supplementary material for: Validation of a self-administered web-based 24-hour dietary recall among pregnant women
Source: BMC Pregnancy Childbirth. 2018 Apr 23;18:112. doi: 10.1186/s12884-018-1741-1 (PMC5913813; doi:10.1186/s12884-018-1741-1)
Supplement: Supplementary file 2 — Table S2. Cross-classification of intakes by quartiles and weighted kappa coefficient in the 1st and 3rd trimesters. This additional file presents the proportion of participants whose dietary intakes reported by both tools in the 1st and 3rd trimesters were ranked in the same, adjacent and opposite quartiles. (DOCX 20 kb) [file 12884_2018_1741_MOESM2_ESM.docx]

**Additional file 2: Table S2: Cross-classification of intakes by quartiles and weighted kappa coefficient in the 1^st^ and 3^rd^ trimesters.**

|  | **%** | | | | **Weighed**  **Kappa** |
| --- | --- | --- | --- | --- | --- |
|  | **Same quartile** | **Adjacent quartiles** | **± 1 Quartile apart** | **Misclassification (quartile 1 vs 4)** |  |
| ***1^st^ trimester*** |  |  |  |  |  |
| Energy | 40.0 | 35.0 | 75.0 | 5.0 | 0.28 |
| Carbohydrates | 41.7 | 35.0 | 76.7 | 1.7 | 0.33 |
| Fat | 41.7 | 36.7 | 78.4 | 6.7 | 0.31 |
| Proteins | 41.7 | 38.3 | 80.0 | 1.7 | 0.36 |
| % Carbohydrates | 35.0 | 35.0 | 70.0 | 5.0 | 0.20 |
| % Fat | 23.0 | 43.3 | 66.3 | 10.0 | 0.04 |
| % Proteins | 36.7 | 41.7 | 78.4 | 5.0 | 0.28 |
| Saturated fatty acids | 40.0 | 38.3 | 78.3 | 5.0 | 0.31 |
| Cholesterol | 28.0 | 50.0 | 78.8 | 6.7 | 0.20 |
| Vitamin A | 26.7 | 46.7 | 73.4 | 6.7 | 0.15 |
| Thiamin | 36.7 | 46.7 | 83.4 | 3.3 | 0.33 |
| Riboflavin | 36.7 | 51.7 | 88.4 | 1.7 | 0.39 |
| Niacin | 38.3 | 40.0 | 78.3 | 6.7 | 0.28 |
| VitB6 | 35 | 43.3 | 78.3 | 3.3 | 0.28 |
| Folic Acid | 26.7 | 36.7 | 63.4 | 10.0 | 0.04 |
| Vitamin B12 | 33.3 | 36.7 | 70.0 | 6.7 | 0.17 |
| Vitamin C | 35.0 | 38.3 | 73.3 | 5.0 | 0.23 |
| Vitamin D | 40.0 | 46.7 | 86.7 | 3.3 | 0.39 |
| Magnesium | 41.7 | 36.7 | 78.4 | 3.3 | 0.33 |
| Phosphorus | 41.7 | 45.0 | 86.7 | 1.7 | 0.41 |
| Zinc | 36.7 | 45.0 | 81.7 | 5.0 | 0.31 |
| Iron | 33.3 | 40.0 | 73.3 | 6.7 | 0.20 |
| Calcium | 45 | 33.3 | 78.3 | 3.3 | 0.36 |
| Potassium | 36.7 | 43.3 | 80.0 | 6.7 | 0.28 |
| Sodium | 31.7 | 46.7 | 78.4 | 3.3 | 0.25 |
| Fibres | 40.0 | 35.0 | 75.0 | 5.0 | 0.28 |
| Average | 36.3 | 41.0 | 77.2 | 4.9 | 0.27 |
| ***3^rd^ trimester*** |  |  |  |  |  |
| Energy | 41.7 | 43.3 | 85.0 | 0 | 0.41 |
| Carbohydrates | 41.7 | 48.3 | 90.0 | 1.7 | 0.44 |
| Fat | 38.3 | 45.0 | 83.3 | 5.0 | 0.33 |
| Proteins | 43.3 | 38.3 | 81.6 | 5.0 | 0.36 |
| % Carbohydrates | 50.0 | 35.0 | 85.0 | 5.0 | 0.44 |
| % Fat | 31.7 | 43.3 | 75.0 | 3.3 | 0.23 |
| % Proteins | 40.0 | 41.7 | 81.7 | 1.7 | 0.36 |
| Saturated fatty acids | 35.0 | 43.3 | 78.3 | 3.3 | 0.28 |
| Cholesterol | 38.3 | 38.3 | 76.6 | 5.0 | 0.28 |
| Vitamin A | 50.0 | 31.7 | 81.7 | 8.3 | 0.39 |
| Thiamin | 40.0 | 46.7 | 86.7 | 3.3 | 0.39 |
| Riboflavin | 46.7 | 36.7 | 83.4 | 0 | 0.44 |
| Niacin | 36.7 | 40.0 | 76.7 | 0 | 0.31 |
| VitB6 | 48.3 | 36.7 | 85.0 | 3.3 | 0.44 |
| Folic Acid | 40.0 | 41.7 | 81.7 | 1.7 | 0.36 |
| Vitamin B12 | 40.0 | 41.7 | 81.7 | 1.7 | 0.36 |
| Vitamin C | 35.0 | 45.0 | 80.0 | 1.7 | 0.31 |
| Vitamin D | 50.0 | 28.3 | 78.3 | 5.0 | 0.39 |
| Magnesium | 46.7 | 45.0 | 91.7 | 1.7 | 0.49 |
| Phosphorus | 48.3 | 36.7 | 85.0 | 0 | 0.47 |
| Zinc | 43.3 | 35.0 | 78.3 | 8.3 | 0.31 |
| Iron | 33.3 | 43.3 | 76.6 | 3.3 | 0.25 |
| Calcium | 43.3 | 46.7 | 90.0 | 0 | 0.47 |
| Potassium | 45.0 | 41.7 | 86.7 | 1.7 | 0.44 |
| Sodium | 41.7 | 41.7 | 83.4 | 5.0 | 0.36 |
| Fibres | 53.0 | 36.7 | 89.7 | 0 | 0.55 |
| Average | 42.4 | 40.5 | 82.8 | 2.9 | 0.38 |
