# Supplementary material for: Validation of a self-administered web-based 24-hour dietary recall among pregnant women
Source: BMC Pregnancy Childbirth. 2018 Apr 23;18:112. doi: 10.1186/s12884-018-1741-1 (PMC5913813; doi:10.1186/s12884-018-1741-1)
Supplement: Supplementary file 3 — Table S3. Seven criteria validity analysis of the R24W in the 1st and 3rd trimesters. This additional file presents a summary of all agreement and association analyses conducted in the 1st and 3rd trimesters. (DOCX 21 kb) [file 12884_2018_1741_MOESM3_ESM.docx]

**Additioanal file 3: Table S3: Seven criteria validity analysis of the R24W in the 1^st^ and 3^rd^ trimesters.**

|  | | Individual level | | | | | Group level | | | | Total of poor outcomes |
| --- | --- | --- | --- | --- | --- | --- | --- | --- | --- | --- | --- |
|  | | Association | | Agreement | | | Agreement | | | Presence of bias |  |
|  | Pearson  coefficient | | Cross-classification | | Kappa score | % difference | | T-test | Bland-Altman | |  |
| Criteria for good outcome (G) | ≥0.50 | | ≥ 50% in same quartile; <10% in opposite quartile | | ≥0.61 | 0-10.9% | | P>0.05 | P>0.05 | |  |
| Criteria for acceptable outcome (A) | 0.20-0.49 | |  | | 0.20-0.60 | 11.0-20% | |  |  | |  |
| Criteria for poor outcome (P) | <0.20 | | <50% in same quartile; ≥10% in opposite quartile | | <0.20 | >20% | | P≤0.05 | P≤0.05 | |  |
| ***1^st^ trimester*** |  | |  | |  |  | |  |  | |  |
| Energy | A | | P-G | | A | G | | P | G | | 2 |
| Carbohydrates | A | | P-G | | A | G | | G | G | | 1 |
| Fat | A | | P-G | | A | G | | P | G | | 2 |
| Proteins | G | | P-G | | A | G | | G | G | | 1 |
| % Carbohydrates | A | | P-G | | A | G | | G | G | | 1 |
| % Fat | P | | P-P | | P | G | | G | G | | 4 |
| % Proteins | A | | P-G | | A | G | | G | G | | 1 |
| Saturated fatty acids | A | | P-G | | A | A | | P | G | | 2 |
| Cholesterol | A | | P-G | | A | G | | G | G | | 1 |
| Vitamin A | A | | P-G | | P | G | | G | G | | 2 |
| Thiamin | A | | P-G | | A | G | | G | P | | 2 |
| Riboflavin | G | | P-G | | A | G | | P | G | | 2 |
| Niacin | A | | P-G | | A | G | | G | G | | 1 |
| VitB6 | A | | P-G | | A | G | | G | G | | 1 |
| Folic Acid | P | | P-P | | P | G | | G | G | | 4 |
| Vitamin B12 | P | | P-G | | P | G | | G | G | | 3 |
| Vitamin C | A | | P-G | | A | G | | G | P | | 2 |
| Vitamin D | G | | P-G | | P | P | | P | G | | 4 |
| Magnesium | A | | P-G | | A | G | | G | G | | 1 |
| Phosphorus | G | | P-G | | A | G | | P | G | | 2 |
| Zinc | A | | P-G | | A | G | | G | G | | 1 |
| Iron | A | | P-G | | A | G | | G | G | | 1 |
| Calcium | G | | P-G | | A | P | | P | G | | 3 |
| Potassium | A | | P-G | | A | G | | G | G | | 1 |
| Sodium | A | | P-G | | A | A | | P | G | | 2 |
| Fibres | G | | P-G | | A | G | | G | G | | 1 |
| Total of poor outcomes | 3 | | 26-2 | | 5 | 2 | | 8 | 2 | | 48 |
| Average |  | |  | |  |  | |  |  | | 1.9 |
| ***3^rd^ trimester*** |  | |  | |  |  | |  |  | |  |
| Energy | G | | P-G | | A | G | | G | G | | 1 |
| Carbohydrates | G | | P-G | | A | G | | G | P | | 2 |
| Fat | A | | P-G | | A | G | | G | G | | 1 |
| Proteins | G | | P-G | | A | G | | G | G | | 1 |
| % Carbohydrates | G | | G-G | | A | G | | G | P | | 1 |
| % Fat | A | | P-G | | A | G | | P | P | | 3 |
| % Proteins | G | | P-G | | A | G | | G | G | | 1 |
| Saturated fatty acids | G | | P-G | | A | A | | P | G | | 2 |
| Cholesterol | G | | P-G | | A | G | | P | G | | 2 |
| Vitamin A | A | | G-G | | A | G | | G | G | | 0 |
| Thiamin | G | | P-G | | A | G | | G | P | | 2 |
| Riboflavin | G | | P-G | | A | G | | G | G | | 1 |
| Niacin | G | | P-G | | A | G | | G | P | | 2 |
| VitB6 | A | | P-G | | A | G | | P | P | | 3 |
| Folic Acid | G | | P-G | | A | G | | G | P | | 2 |
| Vitamin B12 | A | | P-G | | A | G | | G | G | | 1 |
| Vitamin C | G | | P-G | | A | G | | G | G | | 1 |
| Vitamin D | A | | G-G | | A | A | | P | G | | 1 |
| Magnesium | G | | P-G | | A | G | | P | G | | 2 |
| Phosphorus | G | | P-G | | A | G | | P | G | | 2 |
| Zinc | A | | P-G | | A | G | | G | G | | 1 |
| Iron | G | | P-G | | A | G | | G | G | | 1 |
| Calcium | G | | P-G | | A | P | | P | G | | 3 |
| Potassium | G | | P-G | | A | G | | G | G | | 1 |
| Sodium | G | | P-G | | A | G | | G | G | | 1 |
| Fibres | G | | G-G | | A | G | | G | P | | 1 |
| Total of poor outcomes | 0 | | 22-0 | | 0 | 1 | | 8 | 8 | | 39 |
| Average |  | |  | |  |  | |  |  | | 1.5 |
